# Prevalence, outcomes and associated factors of labor induction among women delivered at public hospitals of MEKELLE town-(a hospital based cross sectional study)

**DOI:** 10.1186/s12884-020-02862-7

**Published:** 2020-04-09

**Authors:** Garang Dakjur Lueth, Angesom Kebede, Araya Abrha Medhanyie

**Affiliations:** 1 College of Medicine and Health Sciences, Juba University, Fellow of East, Central and Southern Africa college of Obstetrics and Gynecology, Juba, South Sudan; 2 Obstetrician and Gynecologist, Infertility and ART specialist and Gynecology Laparoscopic surgeon, Ayder comprehensive Specialized Hospital, College of Health Sciences, Mekelle University, Mekelle, Tigray Ethiopia; 3 Global Health and Development, Head, MARCH Research Center and School of Public Health, College of Health Sciences, Mekelle University, Mekelle, Tigray Ethiopia

**Keywords:** Induction of labor, Prevalence, Outcome, Indication, Misoprostol, Ripening, Bishop’s score

## Abstract

**Background:**

Induction of labor refers to iatrogenic stimulation of uterine contractions before the onset of spontaneous labor as a therapeutic option when benefits of expeditious delivery outweigh the risks of continuing the pregnancy. This research was to study the prevalence, outcomes and associated factors of labor induction among women delivered at Ayder comprehensive specialized hospital and Mekelle general hospital in Mekelle town, Tigray, North Ethiopia.

**Methods:**

A hospital based cross sectional study was conducted on 346 laboring mothers who delivered after induction of labor, from January 1st, to July 31st, 2017. Using structured questionnaire and quota sampling techniques, all eligible participants were immediately enrolled upon admission until the desired sample size was achieved. SPSS windows version 23.0 was used for analysis and both descriptive and inferential statistics were conducted; statistical significance to declare relationship between the dependent and independent variables was set at *p* < 0.05.

**Results:**

Total of 3834 women delivered at the study area out of which 346 were induced making (9%) prevalence of induction. Out of this, 244 (70.5%) delivered vaginally, 19 (5.5%) were instrumental deliveries and 83 (24%) by Cesarean section, induction was successful in 263 (76%) while the failure rate was 25 (7.2%).

All who failed induction (25) were delivered by cesarean section making a 3.3% contribution of failed induction into the overall rate of the institutions cesarean deliveries during the study period.

Prolonged rupture of membranes was the commonest indication and Bishop’s score after cervical ripening significantly predicted the success of induction [AOR = 8.150, 95% CI = (1.265, 52.526)].

**Conclusion:**

Our prevalence of labor induction is very low compared to the rate of other institutions in developed countries, rate of successful inductions (76%) is slightly higher than the rate of similar institutions in Ethiopia but comparable to the regional rates while failed induction is very low in comparison to both local and regional institutions. Bishop’s score significantly predicted the success of induction.

## Background

Induction of labor refers to the iatrogenic stimulation of uterine contractions, (before the spontaneous onset of labor), as a therapeutic option when the benefits of expeditious delivery outweigh the risks of continuing the pregnancy [1]. Rates of induction of labor varies from region to region with progressive increase to nearly doubling of incidence in some of the developed countries [2, 3]. In the United States of America and United Kingdom about 20% of all deliveries are by induction of labor, with some institutions going to an incidence of as high as 40%, while 11.4% is reported in Latin America [2, 4] and an average of 4.4% in Africa [5].

Methods for induction of labor may be divided into mechanical and pharmacological both of which have their own advantages and drawbacks [6]. Choice among this methods depends on many factors, including cervical condition, presence or absence of uterine scar, parity, available resources and obstetrician preference [6].

### Statement of the problem

As compared with expectant management, induction of labor (IOL) might be associated with better maternal and perinatal outcomes as well as adverse outcome depending on many factors, despite this outcomes it is the most commonly performed obstetric procedure worldwide [7, 8].

According to WHO secondary analysis study on unmet need for induction of labor, African rates of induction of labor are still very far from the expected, averaging 4.4% and 60–80.2% unmet need for labor induction [4, 5].

Although there is no commonly accepted definition of “failed labor induction.” the current practice acknowledges that “allowing at least 12–18 h of latent labor before diagnosing a failed induction may reduce the risk of cesarean delivery [9]”, Options of management following failed induction of labor include a further attempt to induce labor after consultation with the patient or performing a caesarean section [6], Ethiopian national guideline for induction recommends Cesarean section only after declared failed induction.

Although induction of labor is a daily practice at public and private health institutions in Ethiopia, including the study area, there is a limitation in undertaking a study on prevalence, outcomes and associated factors of labor induction. Most of the studies concentrated on the success or failure rate of labor induction as a sole outcome, despite abundant literature on various outcomes [4, 8, 10].

### Justification of the study

Since Ethiopian demographic health survey database do not include information on induction of labor, this study will stimulate further studies perhaps nationwide to address national rate of induction.

Induction when successful results in vaginal delivery but sometimes fails with potential adverse outcomes including high Cesarean delivery rate, for this reason the current international guidelines allow at least 12–18 h of latent labor before diagnosing failed induction and further attempt to induce labor after consultation with the mother, while Ethiopian national protocol for induction recommends only Cesarean section after declared failed induction, undertaking this study will help us knowing the contribution of failed induction into the overall institutional cesarean section rate as well as the other adverse outcomes. This can be used as evidence that we can build on with more well designed randomized control trials to help the policy makers revising the national guideline.

Knowledge of the outcomes of induction at the institutional level will be employed as a database to monitor the rate, common indications and outcomes for future improvement of the quality of care as well as an evidence based information for counseling of mothers for induction.

## Methods

This study was conducted from January 1st, 2017 to July 31st, 2017 at Ayder comprehensive specialized Hospital (ACSH) and Mekelle General Hospital (MGH) which are public Teaching hospitals located in Mekelle town, the capital city of Tigray national regional state, north Ethiopia, and 789 Kilometers from Addis Ababa, The capital city of Ethiopia.

Both hospitals are used by Mekelle University College of health sciences as teaching institutions; they have seventy four obstetrics beds and annual deliveries of 5000 to 5500. During the study period the Department of Obstetrics and Gynecology had 8 specialists, one subspecialist, 35 residents at different residency levels (year1 – year4), 41 nurse midwifes and variable number of interns.

The study population was pregnant women who voluntarily accepted to participate in the study and admitted after 28th completed weeks for termination of pregnancy by induction of labor as per the national guideline.

The sample size will be calculated using the single population proportion formula:

$$ n=\frac{z_{\alpha /2}^2p\left(1-p\right)}{d^2} $$

Where:

*n* = sample size.

Z = standard normal distribution corresponding to significance level at α = 0.05.

*p* = proportion of labor induction success.

“P”, proportion of labor induction success is assumed to be 65.7% which was derived from study conducted at Jimma University Specialized Hospital [9].

d = margin of error 5%.

$$ n=\frac{(1.96)^2\times 0.657\left(1-0.657\right)}{(0.05)^2}=346. $$

**Figure 1:**
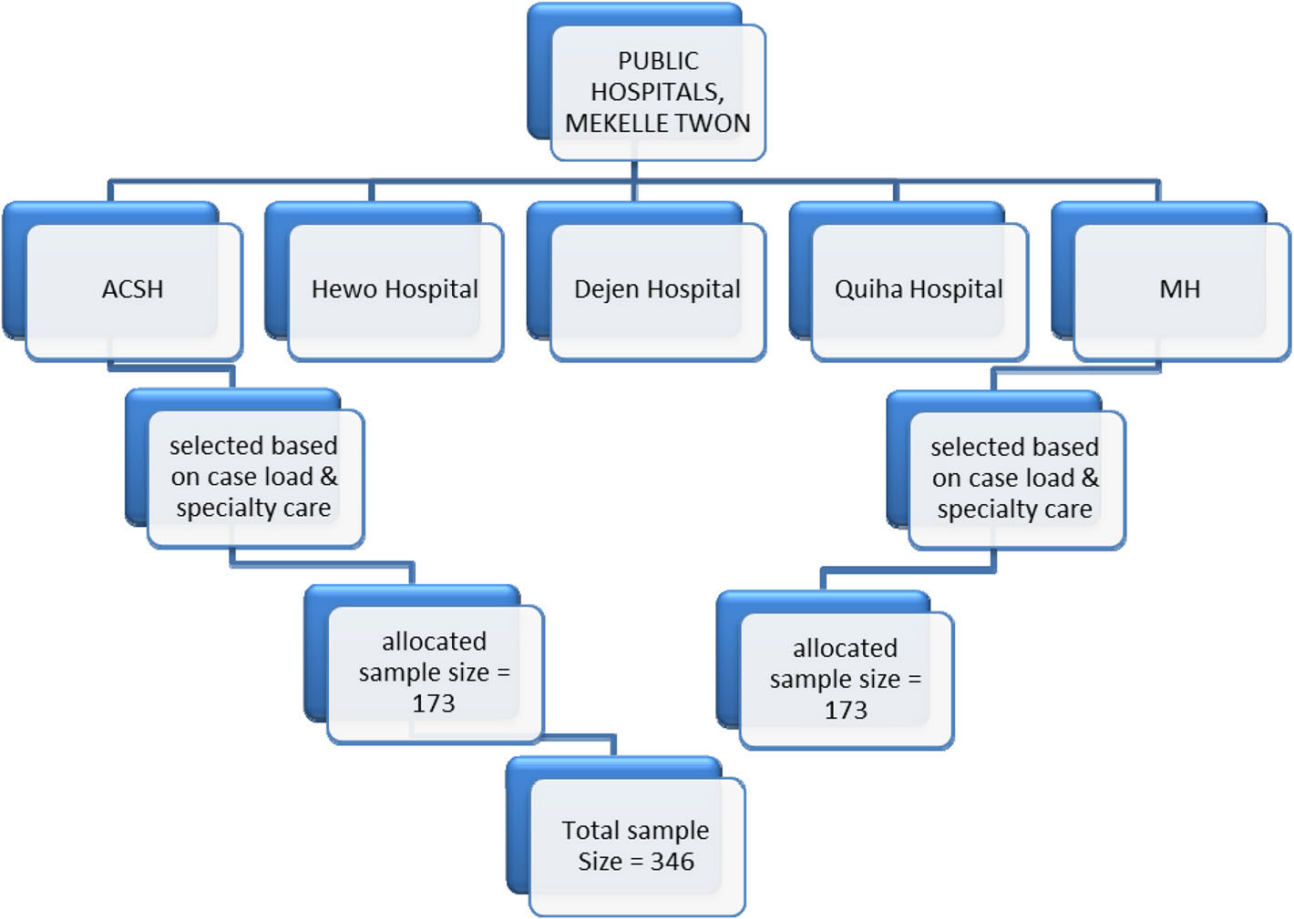
Fig. 1: schematic presentation of sampling procedure.

Since both selected hospitals have the same case load and specialty care, the calculated sample size was equally divided into two giving a sample size of 173 per each hospital (Fig. 1).

After accurate revision of medical records as well as direct interview of patients, using structured questionnaire and quota sampling techniques, all eligible participants were immediately enrolled upon admission until the desired sample size was achieved.

Upon enrollment and through the period of labor and delivery, information regarding **socio-**demographic factors, obstetric history and physical examination findings, indication for induction, methods used for induction and outcomes, was collected within 6 hours after delivery from the medical records files, operating theatre and neonatal intensive care unit records using structured and comprehensive questionnaire designed for that. Oral interview was also used when necessary and missed information was resolved with the managing Physicians before discharge of the mother and her neonate.

One day training was conducted for data collectors (3 midwives and 2 year one residents) to outline the objectives and relevance of the study, who are the eligible participants, procedures of data collection techniques and the whole contents of the questionnaire. The questionnaire was pre- tested by taking 5% of the calculated sample size and necessary modification were made based on the nature of gaps identified. The principal investigator supervised data collectors on daily basis through revision and checking for completeness, accuracy and consistency. Research data were stored in a way that ensured security and confidentiality.

After data collection, each questionnaire was coded and entered in to prepared template of SPSS Version 23; frequency counts were conducted to check for accuracy, consistency and missed values. Age and parity were reclassified to facilitate analysis. The prevalence of labor induction as well as the rate of cesarean deliveries for failed induction was calculated out of the total deliveries during the study period. Descriptive statistics like frequency tables, mean, standard deviation, minimum and maximum, when applicable and descriptive summaries were used to describe the study variables. Associations were tested using chi square or Fisher’s exact test as appropriate; the level of statistical significance was set at *p* < 0.05. Multivariate logistic regression analysis was employed to assess independent determinants of successful induction of labor. Odd ratios (95% confidence intervals) were used to determine the association of different factors with the success of induction.

Ethical clearance to conduct the study was obtained from the Ethics and research committee of Mekelle University, College of Health Sciences [ERC0898/2017].

The aim and purpose of the study was explained to all participants using patient information sheet. Participants were enrolled to the study only when they gave informed and written consent that they are willing to take part in the study.

Study participants were informed about their right not to give response to a question, and their right to withdraw from the study if they felt any discomfort. It was also emphasized that their withdrawal will not affect their management. The participants were followed throughout labor and postpartum without intervention apart from the standard procedures for labor induction as outlined by the national guidelines.

At Ayder Referral hospital both mechanical (balloon catheter) and pharmacological (vaginal misoprostol, oxytocin) methods are employed for induction of labor depending on favorability of the cervix and the indication for induction. With unfavorable cervices, defined by Bishop’s Score less than 4 (Table 1), 25–50 μg vaginal misoprostol given in 6 hours intervals and maximum of 200 μg is the ripening agent used, sometimes, especially in cases of severe oligohydramnios, balloon catheter is used.

**Table 1:** Bishop Score

Score	Dilation	Effacement (%)	Station^a^	Consistency	Position
0	closed	00–30	−3	Firm	posterior
1	1–2	40–50	−2	medium	Mid position
2	3–4	60–70	−1,0	soft	Anterior
3	≥ 5	≥ 80	+ 1, + 2		

^a^Station is graded from −3 to + 3

*Interpretation of the Bishop’s score:*

Score < 4: Unfavorable cervix

Score 5–8: Intermediate

Score ≥ 9: Favorable cervix

Modified from the Ethiopian National Guideline

With the use of misoprostol some women goes into active labor and some require subsequent induction with oxytocin. Oxytocin induction chart was utilized to monitor the maternal vital signs, fetal heart rate, uterine activity, rate of oxytocin infusion and the dose of oxytocin.

Ayder referral hospital is following the national protocol [20] for induction of labor using 5 units of oxytocin in 1000 ml of IV fluids (normal saline in most of the times) for nulliparous, starting by 5mu/min with dose increment every 30 min till adequate uterine contractions are attained (Table 2).

**Table 2:** Schedule for oxytocin dosage escalation

Dose and oxytocin concentration	Time	Drops / minute 1 ml ≈ 20 drops	Approximate oxytocin in mIU/ minute
First dose: 5 IU of oxytocin in 1000 ml fluid	0:00 h	20	2
	0:30 h	40	4
	1:00 h	60	6
	1:30 h	80	8
Second dose: Add another 5 IU of oxytocin to the remaining first dose fluid	2:00 h	50	12
	2:30 h	60	15
	3:00 h	80	20
Third dose: Add another 5 IU of oxytocin on the remaining second dose fluid	3:30 h	50	24
	4:00 h	60	30
	4:30 h	80	40
	5:00 h	As above	As above
	5:30 h	As above	As above

## Results

During the study period, a total of (3834) woman delivered at the study area, out of which (346) were induced making a prevalence of induction of (9%).Total number of cesarean deliveries in this period were 770, out of which 25 were done for failed induction of labor making 3.3% contribution of failed induction into the overall rate of Cesarean deliveries during the study period.

### Sociodemographic characteristics of the participants

Ages of participants ranged from 16 to 44 years with mean age of (26.8 ± 5.0) years. They were almost Tigriyans by Ethnicity 341(98.6%) with few participants from Amhara, 2(0.6%) and Afar 3(0.9%). One hundred twelve (32.4%) were from the rural areas while 234(67.6%) were form urban areas. Looking into their educational background, 36(10.4%) were uneducated, 104 (30.1%) had primary Education, 111(32.1%) secondary education and 95 (27.5%) tertiary education. Occupation wise almost half of the respondents were house wives 170(49.1%), 125 (36.1%) civil servants, another 42(12.1%) were self-employed and 9(2.6%) were students.

The mean family monthly income of the participants was 4169.4 Ethiopian Birr, a maximum income of 14,000 and a minimum income of 700 with standard deviation of 2130. (Table 3):

**Table 3:** Sociodemographic characteristics of the participants (*N* = 346)

Variables		NO	Percentage
Age in Years	Mean (SD)	26.8 (5.0)	–
	< 20	7	2.0
	20–34	310	89.6
	≥ 35	29	8.4
Ethnicity	Tigray	341	98.6
	Amhara	2	0.6
	Afar	3	0.9
Place of residence	Rural	112	32.4
	Urban	234	67.6
Participant’s level of education	Uneducated	36	10.4
	Primary	104	30.1
	Secondary	111	32.1
	Tertiary Education	95	27.5
Participants occupation	Student	9	2.6
	Self employed	42	12.1
	Civil Servant	125	36.1
	House wife	170	49.1
Average family monthly income	Mean + SD	4169.4 + 2130	
	Minimum	700	–
	Maximum	14,000	
	Mean	4169.4	

### Obstetrics and physical examination finding characteristics

Half of the participants were primipara 181(52.3%), while 38(11%) were grand multipara and the rest were ranging from Para two to four 127(36.7%). Two hundred twenty five (65.0%) were term by Gestational age at admission, 78(22.5) were postterm and 43(12.4%) were Preterm and 28(8.1%).

Nearly half of the ANC follow up were at public hospitals 171(49.4%), 124(35.8%) at health centers, and 50(14.5%) at private Hospitals and Clinics, while only one participant had no ANC follow up. Most of the participants were healthy with no medical illnesses 338(97.7%), 6(1.7%) were known to have chronic hypertension, 1(0.3%) diabetic, and 1(0.3%) was known to have cardiac disease.

Prolonged rupture of membranes was the most common indication for induction of labor 143(41.3%), followed by post-term pregnancy 68(19.7%), hypertensive disorders 57 (16.5%), oligohydramnios 29(8.4%), IUFD 26(7.5%), APH 18(5.2%), congenital malformations 3(0.9%) and IUGR 2(0.6%). Before induction of labor 10(2.9%) of the fetuses were diagnosed to have malformations, Anencephaly being the most common malformation 5 (50%), followed by 3 (30%) ventriculomegally and one case of achondroplasia.

On physical examination, 232(67.1%) were found to have unfavorable Bishop’s Score, while 114(32.9%) had favorable bishop score (Table 4).

**Table 4:** Obstetrics and physical examination findings characteristics of the participants (*N* = 346)

Variables		NO	Percentage
Parity	Para I	181	52.3
	Para II - IV	127	36.7
	Para V& above	38	11
Gestational age	Preterm	43	12.4
	Term	225	65.0
	Postterm	78	22.5
ANC follow up	Health Centre	124	35.8
	Public Hospital	171	49.4
	Private Hospital/ Clinic	50	14.5
	No ANC follow up	1	0.3
Known medical illness	Hypertension	6	1.7
	Diabetes	1	0.3
	Cardiac Disease	1	0.3
	None	338	97.7
Indication for induction	Postterm	68	19.7
	PROM	143	41.3
	APH	18	5.2
	Hypertensive Disorders	57	16.5
	IUFD	26	7.5
	Oligohydramnios	29	8.4
	IUGR	2	0.6
	Malformation	3	0.9
Status of the fetus before induction	Dead	32	9.2
	Alive	314	90.8
Any diagnosed Malformation	Yes	10	2.9
	No	336	97.1
Specific malformation	Anencephaly	5	1.4
	Ventriculomegally	3	0.9
	Achondroplasia	1	0.3
	Club Foot	1	0.3
Bishop’s score at Admission	Favorable	114	32.9
	Unfavorable	232	67.1

### Methods of ripening and induction of labor

Cervical ripening was done for 225 (65%) (those with unfavorable Bishop’s Score), misoprostol was the most common method of ripening 195(86.7%) while only 30 (13.3%) participants were ripened by Balloon Catheter. After cervical ripening 217 (96.4%) of the participants had favorable Bishop, but 8(3.6%) were still with unfavorable Bishop’s score. Oxytocin infusion was the common method of induction in about two thirds of the Participants 214 (61.8%) and 132 (38.2%) were induced by misoprostol. For those who were initiated on oxytocin infusion, 81 (37.9%) were maintained at the second dose, 58 (27.1%) at the third and maximum dose and 17 (7.9%) at the first dose.

Mean induction to delivery time was 8.9 h, maximum of 22 h and a minimum of 1 h (Table 5).

**Table 5:** Methods of cervical ripening and induction

Variables		No	Percentage
Cervical ripening	Yes	225	65
(*N* = 346)	No	121	35
Ripening agent	Misoprostol	195	86.7
(*N* = 225)	Balloon catheter	30	13.3
Bishop’s score after cervical ripening	Favorable	217	96.4
*N* = (225)	Unfavorable	8	3.6
Method of induction	Oxytocin	214	61.8
	Misoprostol	132	38.2
Maintenance Dose of Oxytocin (*N* = 214)	First dose	17	7.9
	Second dose	81	37.9
	Third dose	58	27.1
	Maximum dose	58	27.1
Induction to delivery time in hours	Mean	8.9	
	Median	8.0	
	Range	21.0	–
	Minimum	1.00	
	Maximum	22.0	

### Outcomes of labor induction

#### Birth outcomes

Out of the total number of induced mothers, 244 (70.5%) delivered spontaneously, 19 (5.5%) were assisted by instrumental delivery and 83 (24%) by Cesarean section. By excluding 58 cases that had cesarean section for reasons other than failed induction, induction was successful in 263 (76%) and the failure rate was 25 (7.2%).

Out of those who were delivered by cesarean section, 25(30.1%) were done for failed induction, Other indications for cesarean section were, NRFHRP 41(49.4%), CPD 14(16.9%), Intrapartum APH 2 (2.4%) and secondary arrest 1(1.2%).

One hundred eighty two (52.6%) of the outcomes were males and 164(47.4%) were females, almost half of the neonatal birth weight 180(52%) ranged from 2.5 to 3.4 Kg, 111(32.1%) were ≥ 3.5 and 55(15.9%) were below the 2.5 kg (Table 6).

**Table 6:** Birth outcomes of induced mothers

Birth outcomes			
Variables		**No**	**Frequency**
Mode of delivery (*N* = 346)	Vaginal	244	70.5
	Instrumental	19	5.5
	Cesarean	83	24.0
Failure/success of induction (*N* = 346)	Successful Induction	263	76
	Failed Induction	25	7.2
Indications for Cesarean delivery (*N* = 83)	NRFHRP	41	49.4
	CPD	14	16.9
	Failed Induction	25	30.1
	Intrapartum APH	2	2.4
	Secondary Arrest	1	1.2
Outcome (*N* = 346)	Male	182	52.6
	Female	164	47.4
5th Minutes APGAR score (*N* = 312)	> 7	276	88.5
	< 7	36	11.5
Birth weight (*N* = 346)	< 2.5	55	15.9
	2.5–3.4	180	52.0
	> 3.5	111	32.1

### Maternal peripartum complications

Some of the observed adverse maternal outcomes of induction were 15(4.3%) PPH, 7 (2%) precipitated labor, 5 (1.4%) intrapartum placental abruptions, 3(0.9%) incomplete uterine ruptures, 1(0.3%) uterine over activity and 1 (0.3%) maternal death.

The only maternal death in this study was a multiparous woman that was induced by oxytocin and delivered successfully vaginally. After delivery she developed refractory postpartum hemorrhage secondary to uterine atony and measures including bimanual compression of the uterus, B-Lynch and finally total abdominal hysterectomy were done. She was admitted and died in the ICU the second day (Table 7).

**Table 7:** Maternal peripartum complications of induced mothers

Maternal peripartum complications			
Variables		**No**	**Percentage**
Uterine over activity	Yes	1	0.3
	No	345	99.7
Precipitated labor	Yes	7	2.0
	No	339	98.0
Placental abruption	Yes	5	1.4
	No	341	98.6
Uterine rupture	Yes	3	0.9
	No	343	99.1
Postpartum Hemorrhage	Yes	15	4.3
	No	331	95.7
Maternal death	Yes	1	0.3
	No	345	99.7

### Fetal/ neonatal peripartum complications

In regard to fetal /early neonatal complications, 62 (17.9%) of the neonates had NRFHRP, 2 (0.6%) IUFD, 34 (10.9%) low Apgar score, 10 (3.2%) Early onset neonatal Sepsis and out of this 37 were admitted to the neonatal intensive care unit and half of them stayed for 4–7 days at the NICU and 4 neonates died within the first 7 days of life. Two of the four early neonatal deaths were due to Hyaline membrane disease secondary to prematurity, one due to congenital malformation and another one for perinatal asphyxia (Table 8).

**Table 8:** Peripartum complications of fetus/neonates delivered by induction

Fetal / Neonatal peripartum complications			
Variables		**No**	**Percentage**
NRFHRP	Yes	62	17.9
	No	284	82.1
IUFD	Yes	2	0.6
	No	344	99.4
Low APGAR score (*N* = 312)	Yes	34	10.9
	No	278	89.1
Admission to NICU	Yes	37	11.9
	No	275	88.1
NICU admission Diagnosis (*N* = 37)	PNA	19	51.4
	EONS	10	27.0
	HMD	4	10.8
	MAS	4	10.8
Duration of stay in NICU (*N* = 37)	24–72 Hours	20	54.1
	4–7 days	17	45.9
EONS	Yes	10	3.2
	No	302	96.8
END	Yes	4	1.3
	No	308	98.7
Cause of END (*N* = 4)	PNA	1	25.0
	HMD	2	50.0
	Malformation	1	25.0

### Association of selected factors to the outcome of induction

#### Cross tabulation

Using the chi-square test value, Maternal age, Residence, parity, gestational age whether the baby was alive or not before induction, Bishop score at admission, whether cervical ripening was done or not, the ripening agent used, bishop’s score after ripening, Method of induction, outcome, birth weight, 5th minutes Apgar score, Neonatal resuscitation and admission to NICU were selected and analyzed for their possible association with the outcome of induction. It was found that parity (*P* value = 0.006), Bishop’s score after cervical ripening (*P* value = 0.001) and Method of induction (*P* value = 0.000) are the only significant variable (Tables 9, 10 and 11).

**Table 9:** Obstetric characteristics in relation to the outcome of induction

Variables		Successful induction	Failed Induction	Total	*P* Value
Age	< 20	5 (100%)	0 (0.0%)	5 (100%)	0.813
	20–34	236 (91.5%)	22 (8.5%)	258 (100%)	
	≥35	22 (88.0%)	3 (12.0%)	25 (100%)	
Residence	Rural	89 (95.7%)	4 (4.3%)	93 (100%)	0.076
	Urban	174 (89.2%)	21 (10.8%)	195 (100%)	
Gravidity	Primigravida	124 (86.7%)	19 (13.3%)	143 (100%)	0.006
	Multigravida	139 (95.9%)	6 (4.1%)	145 (100%)	
Gestational Age	Preterm	33 (89.2%)	4 (10.8%)	37 (100%)	0.868
	Term	169 (91.4%)	16 (8.6%)	185 (100%)	
	Postterm	61 (92.4%)	5 (7.6%)	66 (100%)	
Fetal status before induction	Dead	29 (93.5%)	2 (6.5%)	31 (100%)	0.754
	Alive	234 (91.1%)	23 (8.9%)	257 (100%)	
Bishop’s score at admission	Favorable	95 (93.1%)	7 (6.9%)	102 (100%)	0.517
	Unfavorable	168 (90.3%)	18 (9.7%)	186 (100%)	
Any Cervical Ripening done	Yes	166 (91.2%)	16 (8.8%)	182 (100%)	1.00
	No	97 (91.5%)	9 (8.5%)	106 (100%)	
Ripening Agent	Misoprostol	150 (92.6%)	12 (7.4%)	162 (100%)	0.081
	Balloon Catheter	16 (80.0%)	4 (20.0%)	20 (100%)	
Bishop’s score after Ripening	Favorable	164 (93.2%)	12 (6.8%)	176 (100%)	0.001
	Unfavorable	2 (33.3%)	4 (66.7%)	6 (100%)	
Method of induction	Oxytocin	153 (86.0%)	25 (14.0%)	178 (100.0%)	0.000
	Misoprostol	110 (100.0%)	0 (0.0%)	110 (100.0%)

**Table 10:** Maternal peripartum complications in relation to the outcome of labor induction

Variables		Successful induction	Failed induction	Total	*P* Value
Precipitated labor	Yes	7 (100%)	0 (0.0%)	7 (100%)	0.642
	No	256 (91.1%)	25 (8.9%)	281 (100%)	
Placental abruption	Yes	1 (100%)	0 (0.0%)	1 (100%)	1.000
	No	262 (91.3%)	25 (8.7%)	287 (100%)	
Uterine rupture	Yes	0 (0.0%)	1 (100%)	1 (100%)	0.087
	No	263 (91.6%)	24 (8.4%)	287 (100%)	
PPH	Yes	14 (100%)	0 (0.0%)	14 (100%)	0.383
	No	249 (90.9%)	25 (9.1%)	274 (100%)	
Maternal death	Yes	1 (100.0%)	0 (0.0%)	1 (100%)	1.000
	No	262 (91.3%)	25 (8.7%)	287 (100%)	
Outcome	Male	142 (92.2%)	12 (7.8%)	154 (100%)	0.672
	Female	121 (90.3%)	13 (9.7%)	134 (100%)	
Birth weight	< 2.5	40 (93.0%)	3 (7.0%)	43 (100%)	
	2.5–3.4	135 (90.6%)	14 (9.4%)	149 (100%)	0.883
	≥3.5	88 (91.7%)	8 (8.3%)	149 (100%)	
5th Minutes APGAR score	> 7	216 (91.1%)	21 (8.9%)	237 (100%)	1.000
	< 7	16 (88.9%)		2 (11.1%)

**Table 11:** peripartum fetal complications in relation to the outcome of labor induction

Variables		Successful induction	Failed induction	Total	*P* Value
NRFHRP	Yes	18 (90.0%)	2 (10%)	20 (100%)	1.000
	No	245 (91.4%)	23 (8.6%)	268 (100%)	
IUFD	Yes	2 (100%)	0 (0.0%)	2 (100%)	1.000
	No	261 (91.3%)	25 (8.7%)	286 (100%)	
Low APGAR score (*N* = 312)	Yes	13 (92.9%)	1 (7.1%)	14 (100%)	1.000
	No	219 (90.9%)	22 (9.1%)	241 (100%)	
Admission to NICU	Yes	214 (91.1%)	21 (8.9%)	235 (100%)	1.00
	No	18 (90.0%)	2 (10.0%)	20 (100%)	

### Logistic regression

On Bivariate analysis, parity, Bishop’s score after cervical ripening and Method of induction are the only variables that showed statistical significance, and Multiparous women were three times more likely to succeed induction than the primipara ones [COR = 3.550, 95% CI = (1.374,9.172)]. Women with favorable Bishop’s score after cervical ripening were twenty seven times more likely to Succeed induction than those with unfavorable Bishop Score [COR = 27.3, 95% CI = (4.538, 164.652)]. Those who had induction with Misoprostol were more likely to succeed induction than the Oxytocin group [COR = 0.000, 95% CI = (0.000, 0.009)].

On Multivariate regression (variables that showed statistical significance in bivariate analysis were entered for Multivariate regression), only Bishop Score after cervical ripening persisted as significant predictor of successful induction and mothers with favorable Bishop’s score after cervical ripening were eight times more likely to succeed induction than those with unfavorable Bishop’s score. [AOR = 8.150, 95% CI = (1.265, .52.526)]. Table 12.

**Table 12:** predictors of successful induction of labor

Variables		COR(95% CI)	AOR(95% CI)
Parity	Primipara		1.958(.603,6.358)
	Multipara	3.550 (1.374,9.172)^*^	
Bishop’s score after ripening	Favorable	27.333 (4.538,164.652)^*^	8.150 (1.265, 52.526)^*^
	Unfavorable		
Method of induction	Oxytocin	0.000 (0.000, 0.009)^*^	0.000 (0.000, −-)
	Misoprostol		

NB: * = significance at *P* value < 0.05

## Discussion

In this study the prevalence of labor induction was found to be 9%, while successful induction constituted 76%, failed induction represented 7.2% with 3.3% contribution of failed induction into the overall institutional rate of cesarean deliveries during the study period. The most common indication for induction was prolonged rupture of membranes; Bishop’s score after cervical ripening was a significant predictor of successful induction.

The rate of labor induction (9%) was found to be slightly higher than other studies done at two teaching hospitals in Addis Ababa (4%) [11]. Although it is not a nationwide study, it is also higher than the average rate of induction in Africa (4.4%) ranging from (1.4%) in Niger to [6.8%] in Algeria and lower than the average rates in Asia (12.1%), ranging from (2.5%) in Cambodia to 35.5% in Sri Lanka [5]. This rate was found to be comparable with study done at Nigerian catholic maternity Hospital [12].

Induction of labor is directly relevant to the health related millennium development and sustainable goals (MDGs &MSGs), Given the increasing attention to reducing perinatal and maternal morbidity and mortality, rates of induction of labor have continued to rise over the past few decades specially in the developed countries, the issue that has contributed a lot in the reduction of their maternal and perinatal mortality and morbidity.

Induction rate of 9% reported in this study is comparable to the rate of some institutions in developed countries about thirty years back, confirming that our rate is still very low. Reasons for this low rate might be due to variation in practice since our guidelines don’t allow for elective induction, induction after cesarean section or high unmet need for induction.

Cesarean delivery is one of adverse maternal outcomes of labor induction specially when it fails, the contribution of failed induction into the overall rate of cesarean deliveries in this study (3.3%) is relatively small and this can be explained by the low failure rate of labor induction (7.2%) compared to other studies done in similar institutions in Ethiopia. In Hawassa Public Hospital (17.3%) [13], Jimma University specialized hospital the failure rate was (21.4%) [14], Addis Ababa Teaching Hospital (28.4%) [11] and Addis Ababa Army Hospital (40.3%) [15].

Successful inductions were 76% of all inductions and goes to 91.3% if we exclude the other cesarean deliveries done for indications other than failed induction; this rate is slightly higher than the studies of Addis Ababa Army Hospital (59.7%) [15], Jimma University Hospital (65.7%) [14], Hawassa (61.6%) [13]. And it is comparable (75.9%) to a study done at Nigerian Catholic Maternity Hospital [12]. These variation can be due to the use of Oxytocin infusion as the most common Method of induction in these local institutions, while titrated oral misoprostol was used as one of the methods of induction in our study area as well as the Nigerian study, explaining the comparability to this studies [12]. Similar to many other local and regional institutions [5, 13–16], prolonged rupture of membranes, 143(41.1%) was the most common indication for labor induction, while elective induction was the most common indication in Asia [5]. This can be explained by the commonly shared risk factors for prolonged rupture of membranes both locally and regionally, and the difference in practice guidelines that allow elective induction as in Sri Lanka.

Bishops score after cervical ripening was found to be the only predictor of successful induction in this study hence induction is eight times more likely to succeed if the Bishop’s score is favorable, [AOR =8.150, 95% CI = (1.265,52.526)], unlike other studies that found age, parity and Bishop’s score at admission [14, 15]. This might be due to the wide confidence interval having more successful inductions and few who had failed.

As a limitation of this study, cross sectional studies are of low level of evidence in comparison to other study designs and they cannot be taken as strong evidence to change practice. Another limitation was that Assessment of Bishop’s score was done by more than one resident throughout the study period making it difficult to assess its precision.

## Conclusion

The study revealed the prevalence of induction of labor to be 9%, which is very low in comparison to the rate of other institutions in developed countries. The rate of successful inductions (76%) is slightly higher than the rate of similar institutions in Ethiopia but comparable to the regional rates, while the rate of failed induction (7.2%) is very low in comparison to both local and regional settings. Like many local and regional Hospitals, prolonged rupture of membranes was the most common indication for induction.

Bishop’s score after cervical ripening significantly predicted success of induction and induction was eight times more likely to succeed if the Bishop’s score was Favorable, [AOR = 8.150, 95% CI = (1.265, 52.526)].

## Data Availability

The datasets used and/or analyzed during this study are available from the corresponding author on reasonable request.

## Abbreviations

ANC: Antenatal Care
ACSH: Ayder Comprehensive Specialized Hospital
APGAR: Apgar score
APH: Antepartum Hemorrhage
CHS: College of Health Sciences
C/S: Cesarean Section
END: Early Neonatal Death
EONS: Early onset Neonatal Sepsis
FHR: Fetal Heart Rate
HMD: Hyaline Membrane Disease
IOL: Induction of Labor
IUFD: Intrauterine Fetal Death
IUGR: Intrauterine Growth Restriction
MGH: Mekelle General Hospital
NICU: Neonatal Intensive Care Unit
NRFHRP: Non-Reassuring Fetal Heart Rate Pattern
PPH: Postpartum Hemorrhage
PROM: Prolonged rupture of membranes
SPSS: Statistical Package for Social Sciences
WHO: World Health Organization
